# Adolescents may accurately self-collect pharyngeal and rectal clinical specimens for the detection of *Chlamydia trachomatis* and *Neisseria gonorrhoeae* infection

**DOI:** 10.1371/journal.pone.0255878

**Published:** 2021-09-27

**Authors:** Gabriella Vavala, Cameron Goldbeck, Claire C. Bristow, Chrysovalantis Stafylis, Paul C. Adamson, Dianna Polanco, Manuel A. Ocasio, Jasmine Fournier, Adriana Romero-Espinoza, Risa Flynn, Robert Bolan, M. Isabel Fernandez, Dallas Swendeman, W. Scott Comulada, Sung-Jae Lee, Mary Jane Rotheram-Borus, Jeffrey D. Klausner, the Adolescent Medicine Trials Network (ATN) CARES Team

**Affiliations:** 1 Division of Infectious Diseases, David Geffen School of Medicine, University of California Los Angeles, Los Angeles, CA, United States of America; 2 Department of Psychiatry and Biobehavioral Sciences, David Geffen School of Medicine, University of California Los Angeles, Los Angeles, CA, United States of America; 3 University of California San Diego, San Diego, CA, United States of America; 4 Department of Population and Public Health Sciences, Keck School of Medicine, University of Southern California, Los Angeles, CA, United States of America; 5 Department of Pediatrics/Section of Adolescent Medicine, Tulane University School of Medicine, NewOrleans, LA, United States of America; 6 Los Angeles LGBT Center, Los Angeles, CA, United States of America; 7 Nova Southeastern University, Fort Lauderdale, FL, United States of America; Institut Pasteur de Madagascar, MADAGASCAR

## Abstract

**Background:**

The COVID-19 pandemic illuminated the benefits of telemedicine. Self-collected specimens are a promising alternative to clinician-collected specimens when in-person testing is not feasible. In this study, we assessed the adequacy of self-collected pharyngeal and rectal specimens for the detection of *Chlamydia trachomatis* and *Neisseria gonorrhoeae* among individuals undergoing chlamydia and gonorrhea screening.

**Methods:**

We used data from a large cohort study that included male and female adolescents between the ages of 12–24 years. We considered self-collected specimens adequate for clinical use if the human synthase gene (a control target of the assay) was detected in the specimen.

**Results:**

In total, 2,458 specimens were included in the analysis. The human synthase gene was detected in 99.2% (2,439/2,458) of all self-collected specimens, 99.5% (1,108/1,114) of the pharyngeal specimens, and 99.0% (1,331/1,344) of the rectal specimens.

**Conclusion:**

Self-collected pharyngeal and rectal specimens demonstrated a very high proportion of human gene presence, suggesting that self-collection was accurate. A limitation of this study is that the sample adequacy control detects the presence or absence of the human hydroxymethylbilane synthase gene, but it does not indicate the specific anatomic origin of the human hydroxymethylbilane synthase gene. Self-collected specimens may be an appropriate alternative to clinician-collected specimens.

## Introduction

The COVID-19 pandemic disrupted in-person clinic-based testing. As physicians switch from in-person visits to virtual visits, self-collected specimens are a promising alternative to clinician-collected specimens [1].

Prior studies demonstrate that self-collection of pharyngeal and rectal specimens for the detection of infectious diseases is feasible and acceptable. In a study by Van der Helm *et*. *al*., over 94% of participants indicated that they would self-collect rectal specimens again, and around half of the participants indicated that they preferred self-collected rectal specimens over clinician-collected rectal specimens [2]. Similarly, after self-collection, Wayal *et*. *al*. found that 82% of participants were willing to self-collect their rectal specimen and that 76% were willing to self-collect their pharyngeal specimen in the future [3].

Additionally, prior research compared self-collected specimens to clinician-collected specimens and demonstrated high agreement between positive results [2,4–7]. Some of those studies tested for *C*. *trachomatis* and *N*. *gonorrhoeae* with pharyngeal and rectal specimens on various nucleic acid amplification devices. Some studies of self-collected specimens had more positive *C*. *trachomatis* and *N*. *gonorrhoeae* results than simultaneously clinician-collected specimens, demonstrating sufficient sample collection with self-collected specimens [2,4–7].

While a majority of the self-collection research focuses on sexually transmitted infections, numerous studies asses the adequacy of self-collected specimens for the detection of other infections, including *Streptococcus* and SARS-CoV-2 [8,9]. A study by Murray *et*. *al*. found 94% overall concordance between self-collected pharyngeal specimens and health care worker-collected pharyngeal specimens for the detection of *Streptococcus* [9]. Additionally, Tan *et*. *al*. found 83.6% sensitivity for self-collected pharyngeal SARS-CoV-2 specimens [8].

We sought to determine adequacy of the self-collection of pharyngeal and rectal specimens using assay data from the Xpert® CT/NG assay.

## Materials and methods

We conducted a sub-study to determine the performance of self-collected specimens as part of a larger randomized control trial aimed to evaluate the ability of HIV prevention continuums to increase viral suppression and prevent HIV-infection among youth aged 12–24 years.

### Ethics

The University of California Los Angeles Institutional Review Board (UCLA IRB #16–001674-AM-00006) and Tulane University Review Board (Tulane IRB #1033876) approved the study protocol and consent procedures. Any protocol deviations or indications of adverse events were reported to the Institutional Review Board. The study was registered at ClinicalTrials.gov on April 28, 2017 (#NCT03134833).

### Human subjects

Study staff obtained written informed assent from participants 12–14 years of age and obtained verbal consent from participants 15–24 of age. Additional parental or guardian consent was not required.

### Study population

From May 2017 to September 2019, the Adolescent Medicine Trials Network CARES study interviewers recruited and enrolled male and female (sex assigned at birth) adolescents 12–24 years of age who were either at high risk for Human Immunodeficiency Virus (HIV) infection or HIV-infected and residing in Los Angeles, California or New Orleans, Louisiana. The full study design and enrollment details have been described elsewhere [10,11].

### Specimen collection

Trained study interviewers verbally instructed participants on how to perform self-collection of pharyngeal and rectal specimens. For the initial study visit, the study interviewers used a Fleshlight model (Q Toys, Austin, TX) to demonstrate the self-collection of rectal specimens. They also provided an image to show the acceptable level of fecal contamination on the swab. For pharyngeal swabs, study interviewer used a mirror to locate their own tonsils and demonstrated the self-collection of pharyngeal specimens. The participants self-collected their specimens in private during their study visit. After specimen collection, study participants inserted the swab into the transport tube and secured the top.

The study used the Xpert® Swab Specimen Collection Kit (Cepheid, Sunnyvale, CA) for pharyngeal and rectal specimens. The Xpert® Swab Specimen Collection Kit includes one Copan flock swab (Copan Flock Technology, Brescia, Italy) and a specimen transport tube with Xpert® swab transport reagent. The trained interviewers tested the specimens on-site within minutes of collection using the Cepheid Xpert® CT/NG assay according to manufacturer’s instructions.

The Xpert® CT/NG is a real-time polymerase chain reaction (PCR) assay that detects *C*. *trachomatis* and *N*. *gonorrhoeae* genomic DNA. Each cartridge contains internal quality controls to ensure proper specimen processing. The sample adequacy control (SAC) is a quality control measure that ensures the clinical specimen contains human DNA by detecting the human hydroxymethylbilane synthase (HMBS) gene. The HMBS gene is a single-copy gene that expresses the HMBS protein in a variety of mammalian tissues [12]. Detection of the HMBS gene increases with inflammation as cell turnover increases. The assay amplifies the HMBS gene, a unique chromosomal gene target, for up to 45 cycles; a test failure result occurs if the HMBS gene is not detected after 45 PCR cycles. Specimen degradation, insufficient mixing of the specimen, and inadequately collected specimens can also result in test failure [13].

### Statistical analysis

We determined specimen adequacy for each anatomic site of collection by calculating the proportion of specimens with a pass result (HMBS gene detected) divided by the summation of pass or fail test results (HMBS gene detected and not detected). We excluded the test results where anatomic site was unknown. We calculated a 95% confidence interval with an assumed exact binomial distribution with a two-sided confidence interval. If the proportion was 100%, we calculated the lower 95% confidence interval (CI) using an exact binomial distribution for the proportion (n-1)/n. All analyses were done using SAS v9.4 (SAS Institute Inc., Cary, North Carolina).

## Results

From May 2017 to October 2019, study participants self-collected 2,458 specimens from rectal and pharyngeal anatomic sites, including multiple samples from individual participants. Overall, specimens were primarily from males (82.7%), with a median age of 21 years (interquartile range, 15–24 years), 5.8% of the pharyngeal and rectal specimens were positive for *C*. *trachomatis*, and 4.9% of the pharyngeal and rectal specimens were positive for *N*. *gonorrhoeae*.

The HMBS gene was detected in 99.2% (2,439/2,458; 95% CI, 98.9%-99.6%) of all specimens. The proportion of HMBS- positive specimens by anatomic site was: 99.5% (1,108/1,114; 95% CI, 98.8%-99.8%) for pharyngeal specimens, and 99.0% (1,331/1,344; 95% CI, 98.4%-99.5%) for rectal specimens (S1 Table).

## Discussion

Using HMBS gene detection to determine adequacy of specimen collection, we found that self-collection of pharyngeal and rectal specimens was accurate among adolescents with HIV infection or whose circumstances limit their ability to protect themselves from HIV infection in Los Angeles and New Orleans. The detection of the human gene HMBS target is a marker of the presence of human tissue in a clinical specimen [13]. We identified the HMBS gene in >99% of all self-collected specimens, which suggests adequacy of the self-collection.

Our findings are similar to prior reports that evaluated specimen adequacy based on the HMBS target. In a study by Dize *et*. *al*., female and male participants mailed in self-collected rectal swabs for *N*. *gonorrhoeae* and *C*. *trachomatis* testing on the GeneXpert® CT/NG assay. Of the 448 self-collected rectal swabs submitted, the GeneXpert® assay detected the HMBS target in 83% of specimens upon initial testing and 89.5% of specimens upon repeat testing [14]. The authors of that study hypothesized that the large number of SAC failures could be attributed to the location and lack of observation of specimen collection. Specifically, study participants collected their specimens at home, and thus the participants could have returned their swabs without performing the self-sampling. A study by Geiger *et*. *al*. tested both rectal and pharyngeal self-collected swabs for *N*. *gonorrhoeae* and *C*. *trachomatis* on the GeneXpert® CT/NG assay. Of the 151 pharyngeal self-collected swabs, 95% of those specimens had a detectable HMBS target. Additionally, of the 144 rectal self-collected swabs, 98% of those specimens had a detectable HMBS target [15].

In a prior study by our group, male participants self-collected pharyngeal and rectal specimens at home, then mailed them to a laboratory for testing on the GeneXpert® CT/NG assay. That study analyzed 145 self-collected rectal specimens and 148 self-collected pharyngeal specimens from males. Of those specimens, 97% had a detectable HMBS target [7]. In our current study, the overall success was similarly high, reaffirming the reliability of self-collection.

Our results should be interpreted in light of the following limitations. First, this study did not directly compare self-collected specimens to clinician-collected specimens. Additionally, the SAC control detects the presence or absence of the HMBS gene, but it does not indicate the specific anatomic origin of the HMBS gene. Also, the study did not evaluate the level of understanding of the specimen collection instructions by the participants, and this might have affected the fidelity of the self-collection process. Lastly, we were unable to analyze specimen adequacy by age and sex due to the way that the data were recorded.

Our findings in context with other reports show that self-collection is an accurate means of rectal and/or pharyngeal clinical specimen collection. Self-collected specimens are an appropriate alternative to clinician-collected specimens for the detection of infection when clinician collection is not feasible.

## Supporting information

S1 Table Specimen adequacy as measured by the proportion of tests with detectable human hydroxymethylbilane synthase gene for self-collected pharyngeal and rectal specimens in adolescents and young adults in Los Angeles and New Orleans. (DOCX)

S1 Data (CSV)
