# Supplementary material for: Adolescents may accurately self-collect pharyngeal and rectal clinical specimens for the detection of Chlamydia trachomatis and Neisseria gonorrhoeae infection
Source: PLoS One. 2021 Sep 27;16(9):e0255878. doi: 10.1371/journal.pone.0255878 (PMC8475974; doi:10.1371/journal.pone.0255878)
Supplement: S1 Table — (DOCX) [file pone.0255878.s001.docx]

**Table: Specimen adequacy as measured by the proportion of tests with detectable human hydroxymethylbilane synthase gene for self-collected pharyngeal and rectal specimens in adolescents and young adults in Los Angeles and New Orleans.**

|  | Pharyngeal | Rectal | All sites |
| --- | --- | --- | --- |
|  | **n (%)** | **n (%)** | **n (%)** |
| **Pass** | 1,108 (99.5%) | 1,331 (99.0%) | 2,439 (99.2%) |
| **Fail** | 6 (0.5%) | 13 (1.0%) | 19 (0.8%) |
| Total | 1,114 | 1,344 | 2,458 |

Pass= HMBS gene detected

Fail= HMBS gene not detected
